# Association between Fecal Bile Acids and Levodopa Response in Patients with Parkinson’s Disease

**DOI:** 10.3390/microorganisms12071432

**Published:** 2024-07-15

**Authors:** Xiaoqin He, Yiqiu Lai, Chengjun Mo, Yi Zhang, Penghui Ai, Shaoqing Xu, Yiwei Qian, Qin Xiao, Xiaodong Yang

**Affiliations:** 1 Department of Neurology and Institute of Neurology, Ruijin Hospital, Shanghai Jiao Tong University School of Medicine, Shanghai 200025, China; 18176329672@163.com (X.H.); mochengjun97@163.com (C.M.); aipenghui@sjtu.edu.cn (P.A.); qyw12344@rjh.com.cn (Y.Q.); 2 Department of Geriatrics, Ruijin Hospital, Shanghai Jiao Tong University School of Medicine, Shanghai 200025, China; shaoqingfx@163.com

**Keywords:** Parkinson’s disease, bile acids, levodopa response, gut microbiome

## Abstract

Levodopa is the mainstay of treatments for Parkinson’s disease (PD), but large heterogeneity exists in patient response. Increasing evidence implicates bile acids (BAs) involved in the pathogenesis of PD. Furthermore, BAs have also participated in drug bioavailability. However, the impact of BAs on levodopa response (LR) has not been investigated. This study evaluated the association between fecal BAs and LR. Levodopa challenge test (LCT) was conducted in 92 PD patients to assess LR. A total of 36 fecal BAs and plasma levodopa concentrations were detected using LC-MS/MS. The difference of BAs between subgroups with bottom and top 30% LR were analyzed and fecal samples from the two groups were collected for metagenomic shotgun analysis. No fecal BAs were significantly correlated with LR, except for chenodeoxycholic acid-3-β-D-glucuronide (CDCA-3-β-glucuronide, R = −0.228, *p*-value = 0.039). We found no significant difference in BAs between subgroups with bottom and top 30% LR. What is more, no significant changes in bacterial species composition related to bile acids metabolism or in the proportional representation of genes encoding known bile acids enzymes were observed between the groups. Overall, our data do not support an association between fecal BAs and levodopa response in PD patients. More precise macro-metabolomic approaches are needed to reveal the potential association between gut microbial interactions and the treatment effect of levodopa.

## 1. Introduction

Parkinson’s disease (PD) is the second most common neurodegenerative disease [1]. There is a complex relationship between the gut and the brain, and numerous evidence revealed that PD might originate in the gut [2]. The gut microbiome may be a target for treatment of PD [3,4]. There are no available therapies that can be completely curable for PD, so symptomatic therapies are particularly important. Levodopa continues to be the mainstay of medical treatment, yet there is a highly inter-individual heterogeneity in the clinical outcome of levodopa, resulting from the fact that levodopa can be metabolized peripherally even with metabolism-related inhibitors [5].

It is well recognized that human genetic variation plays a role in therapeutic outcomes; however, the complexity of microbiota may also limit the prediction of drug response based solely on genomic diversity. Pharmacomicrobiomics, an extension of pharmacogenomics, is an innovative field that focuses on the study of the interplay between gut microbiota and drugs [6]. The human gut microbiome has strong drug-metabolizing activities for some medications. Moreover, the gut microbiome has been shown to impact levodopa efficacy. Small-intestinal bacterial overgrowth (SIBO) and *Helicobacter pylori* (*H. pylori*) might exert a significant impact on the efficacy of levodopa treatment, leading to motor fluctuations [7,8]. Recently, studies have found that gut microbiota, such as *Enterococcus faecalis* (*E. faecalis*) and *Eggerthella lenta*, could metabolize levodopa in different pathways and then impair the clinical response to levodopa [9,10].

Bile acids (BAs), including primary BAs and secondary BAs, are the products of cholesterol metabolism and their synthesis is in part mediated and regulated by the gut microbiota, which interact with nuclear receptors on host drug-metabolizing enzyme machinery, could have the potential to impact drug disposition and pharmacokinetics. Emerging data show that microbiota-derived BAs can interfere with drug biopharmaceutical properties and thereby contribute to drug efficacy, such as by medicating gastrointestinal absorption of encapsulated vitamin K, enhancing the anticancer activity of doxorubicin hydrochloride, influencing the plasma concentration of simvastatin and the treatment response in inflammatory bowel disease [11,12,13,14]. Furthermore, studies in humans and mice have demonstrated the neuroprotection effect of ursodeoxycholic acid (UDCA) and tauroursodeoxycholic acid (TUDCA) in PD. UDCA as a promising mitochondrial rescue compound has shown great promise for the disease-modifying treatment of PD, recently high-dose UDCA has been demonstrated to be safe and well tolerated in early PD [15,16].

Though microbiota-derived BAs have the capacity to influence drug biotransformation, the effect of BAs on clinical response to levodopa has not been reported. Therefore, in this study, we aimed to investigate whether fecal BAs could affect LR assessed by levodopa challenge test (LCT) in patients with PD.

## 2. Materials and Methods

### 2.1. Participants

Participants (PD patients, n = 92) were recruited from the movement disorder clinic at the Department of Neurology, Ruijin Hospital, Shanghai Jiao Tong University School of Medicine. Patients who were diagnosed with PD according to the United Kingdom Parkinson’s Disease Society Brain Bank Clinical Diagnostic Criteria [17] were enrolled. Other inclusion and exclusion criteria were described in the Methods section of the Supplementary Materials. All participants signed the written informed consent forms.

### 2.2. Clinical Features Evaluation and Levodopa Challenge Test (LCT)

The clinical features including Hoehn and Yahr stage, age of onset, disease duration, and anti-PD drugs were collected. The motor phenotype was divided into postural instability and gait difficulty dominant (PIGD) and non-PIGD, as described by Stebbins et al. [18]. Levodopa equivalent daily dosage (LEDD) was calculated using classic methods [19]. The standardized levodopa challenge test (LCT) was performed by movement disorder specialists at the hospital in the morning. Participants were required to withdraw dopaminergic agonists for 36 h and levodopa and other anti-PD drugs for 12 h prior to the tests. The levodopa challenge dosage was calculated as 150% of the usual morning levodopa equivalent dose. The “off-state” was defined as the period when all anti-PD drugs were withheld for at least 12 h, and the best “on-state” was defined as the peak of levodopa benefit in the LCT. The conditions of PD patients were evaluated repeatedly after levodopa administration at a 30-min interval. Time to peak and latency to reach the best “on-state” condition were recorded. The Movement Disorder Society-Unified Parkinson’s Disease Rating Scale Part III (MDS-UPDRS part III) was evaluated in both the “off-state” and best “on-state”.

LR (%) was calculated as follows [20]:

$$\mathrm{Levodopa}\;\mathrm{Response}\;\left(\%\right)=\frac{\mathrm{off}\;\mathrm{state}\;\mathrm{MDS}\;\mathrm{UPDRS}\;\mathrm{III}\;\mathrm{scores}-\mathrm{best}\;\mathrm{on}\;\mathrm{state}\;\mathrm{MDS}\;\mathrm{UPDRS}\;\mathrm{III}\;\mathrm{scores}\;}{\mathrm{off}\;\mathrm{state}\;\mathrm{MDS}\;\mathrm{UPDRS}\;\mathrm{III}\;\mathrm{scores}}\times 100\%$$

### 2.3. Sample Collection

The fresh fecal samples of all participants were collected using the fecal collection containers. Blood samples were collected in the “off-state” condition and the best “on-state” condition after the levodopa challenge test, respectively. Blood samples were centrifuged at 4 °C to obtain plasma. All the fecal and plasma samples were stored at −80 °C prior to detection.

### 2.4. Fecal Bile Acids Analysis

Fecal BAs were measured by liquid chromatography–tandem mass spectrometry (LC-MS/MS) analysis. A weight of 50 mg of feces was added with 400 μL extraction solution (methanol/water = 4:1), ground at 4 °C for 6 min, and then ground with a freezer grinder for 6 min (−10 °C, 50 Hz); the mixed sample was kept in ultrasound (5 °C, 40 KHz) for 30 min, stood for 30 min, and centrifuged at 13,000× *g* for 15 min at 4 °C. Finally, the supernatant was injected into the LC-MS/MS system for analysis. The analysis was performed using ultra-high-performance liquid chromatography coupled with a QTRAP^®^ 6500+ LC-MS/MS System from SCIEX (Framingham, MA, USA). Analyte compounds were separated with a BEH C18 column (150 × 2.1 mm, 1.7 μm; Waters). The mobile phase A consisted of a 0.1% formic acid–water solution and the mobile phase B consisted of a 0.1% formic acid–acetonitrile solution. The solvent gradient changed according to the following conditions for equilibrating the systems: from 0 to 10 min, 25% B to 32% B; from 10 to 26 min, 32% B to 75% B; from 26 to 26.1 min, 75% B to 100% B; from 26.1 to 28 min, 100% B to 100% B; from 28 to 28.1 min, 100% B to 25% B; and from 28.1 to 32 min, 25% B to 25%. The column temperature was maintained at 40 °C. During the period of analysis, all these samples were stored at 4 °C. The following source conditions were used for the mass spectrometer’s operation in electrospray negative ion mode: Curtain Gas (CUR:35); Collision Gas (CAD), Medium; IonSpray Voltage (IS), −4500; Temperature (TEM), 350; Ion Source Gas1 (GS1), 40; and Ion Source Gas2 (GS2), 50. Data were analyzed using the quantitative software OS version 3.1.6 (SCIEX, Framingham, MA, USA).

### 2.5. Plasma Levodopa Concentrations and Microbiome Analysis

Plasma levodopa concentrations were measured by liquid chromatography–tandem mass spectrometry (LC-MS/MS) analysis. Fecal metagenomics from the groups with the lowest and highest tertile LR of PD patients was measured by the whole-metagenome shotgun sequencing carried out on the Illumina HiSeq4000 platform (Illumina Inc., San Diego, CA, USA) at Majorbio Bio-Pharm Technology Co., Ltd. (Shanghai, China). Details of the assay procedure were described in the Methods of the Supplementary Materials.

### 2.6. Statistics Analysis

Quantitative data are shown as mean ± standard deviation (SD) or median (interquartile range). Categorical data are shown as the frequency (percentage). The association between fecal BAs and variables associated with LR was analyzed by the Spearman and partial correlation analysis. Differences between the groups with the lowest and highest 30% LR were analyzed by the Mann–Whitney U test or chi-square test. Analysis of covariance (ANCOVA) was performed for correction in the comparison of the two groups, and the adjusted variables included gender, age, BMI, and clinical characteristics (age of onset, disease duration, Hoehn and Yahr stage, LEDD, motor phenotype, and “off-state” MDS-UPDRS part III scores). Statistical analyses were performed using SPSS software (version 22.0; SPSS Inc., Chicago, IL, USA) and R 4.0.5. The significance levels were set at 0.05 (2-tailed). 

## 3. Results

**Figure 1:**
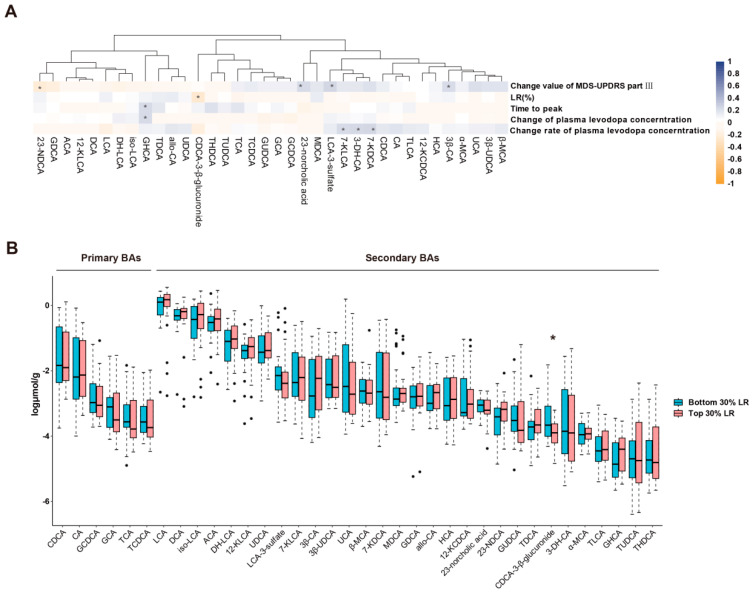
The relationship between fecal BAs and levodopa response. (**A**) The association between 36 fecal BAs and variables of levodopa response in 92 patients with PD. * *p*-value < 0.05. The color of the heat map represented the correlation coefficient. Statistical significance was assessed by Spearman rank correlation analysis. (**B**) The comparison of fecal BAs between the bottom 30% and top 30% LR groups. Abundance is expressed as log μmol per gram of dry-weight feces. * *p*-value < 0.05.

The demographics and clinical characteristics of the study sample are reported in Table 1. We first analyzed the association between fecal BAs and variables related to the response of levodopa in patients with PD (Figure 1A). After adjusting for the influence of other variables by partial correlation analysis, chenodeoxycholic acid-3-β-D-glucuronide (CDCA-3-β-glucuronide) was negatively correlated with LR (R = −0.228, *p*-value = 0.039), glycohyocholic acid (GHCA) was positively correlated with time to peak (R = 0.343, *p*-value = 0.002), and lithocholic acid 3-sulfate (LCA-3-sulfate) was positively correlated with the change value of MDS-UPDRS part III (R = 0.258, *p*-value = 0.019) (Table S1). 

We then compared the differences in fecal BAs between subgroups with the bottom and top 30% LR. However, none of the BAs that has potential correlations with the response of levodopa showed difference between the two groups, despite the concentration of CDCA-3-β-glucuronide being lower in the patients with the top 30% LR than patients with the bottom 30% LR (0.19 ± 0.19 vs. 1.32 ± 3.10 nmol/g, *p*-value = 0.029), and this difference missed the significance after adjustment for age, BMI, gender, and clinical characteristics. Additionally, we did not find any differences in other BAs in the two subgroups (Figure 1B).

**Figure 2:**
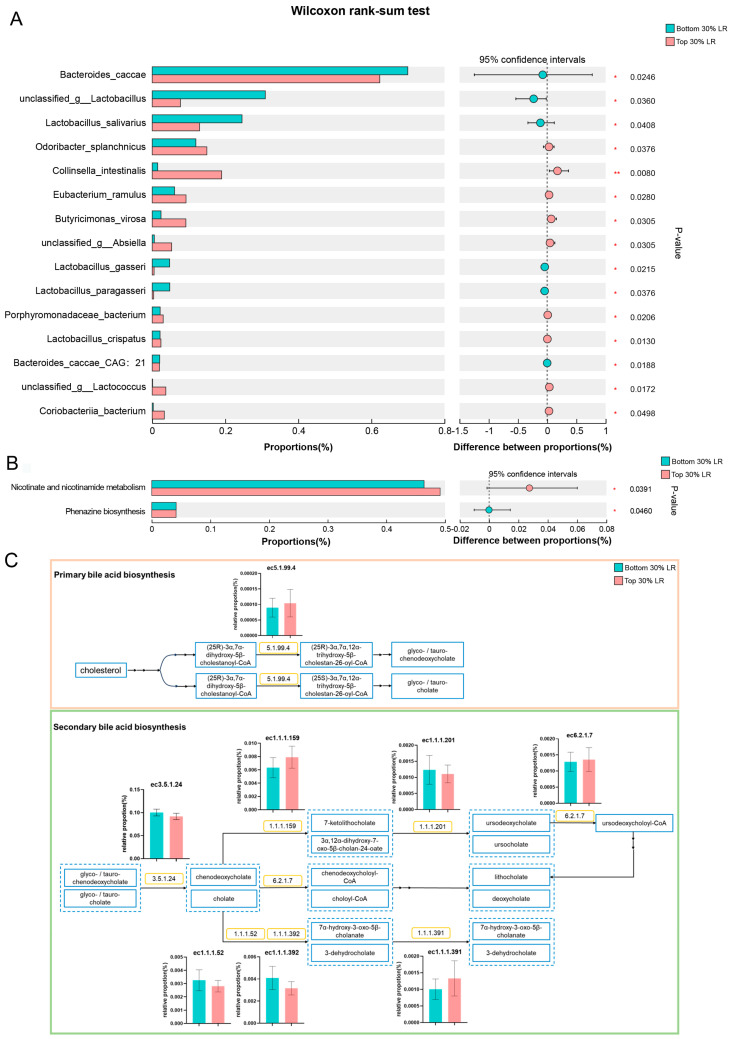
Comparison of subgroups with bottom and top 30% LR shotgun metagenomic sequencing data. Differentially abundant species (**A**) and KEEG level 3 (**B**) between the groups. (**C**) The abundances of microbial gene encoding enzymes involved in primary and secondary bile acids biosynthesis pathways. * *p* < 0.05, ** *p* < 0.01.

Considering the overall species composition, there were no significant differences between the bottom and top 30% LR groups based on alpha- and beta-diversity analyses (Figure S1). Additionally, we identified discriminative bacterial species between the 2 groups: 9 species had significantly higher abundance, while 6 species had significantly lower abundance in the top 30% LR group (Figure 2A and Table S2). However, none of the different gut microbe species were associated with bile acid synthesis and/or metabolism. Functional results (Kyoto Encyclopedia of Genes and Genomes level 3) are shown in Figure 2B and the microbial gene functions related to bile acids biosynthesis pathways showed no difference between the groups (Figure 2C).

## 4. Discussion

This study analyzed the associations between fecal BAs (one important class of microbially produced metabolites) and clinical response to levodopa. The main results demonstrated that the fecal BAs had little relationship with LR.

Levodopa is absorbed into system circulation mainly from the proximal small intestine (jejunum), then enters the brain, and is converted to dopamine to be effective. However, a portion of levodopa is metabolized peripherally, leading to a decrease in striatal dopamine levels and LR reduction [21]. Therefore, it is crucial to elucidate the factors involved in the LR for individuals’ therapeutics in PD. Emerging evidence has revealed that interaction with gut microbiota in the gastrointestinal tract is important for levodopa peripheral metabolism, affecting the treatment outcomes. BAs are now recognized as signaling molecules that affect diverse physiological processes and are also well known to affect drug disposition through effects on absorption and metabolism. The main effect of BAs is their ability to act as both drug-solubilizing and permeation-modifying agents by various mechanisms, including the formation of micelles affecting poorly water-soluble drugs (PWSDs), interaction with biological membranes, inhibition of drug transporters, and competition with drug molecules as substrates [11,22,23]. The catalytic activity of CYP3A4, which is a major cytochrome, P450 (CYP) enzyme-catalyzing substrates may be regulated by BAs [24].

Based on this evidence, we tried to explore whether fecal BAs could influence the therapeutic response to levodopa. However, our results suggested that the fecal BAs had little relationship with levodopa treatment response. There are some possible explanations for these findings. First, levodopa is a water-soluble substance, which is not a PWSD whose absorption increased by forming micelles regulated by gut microbial 7a-dehydroxylation. Secondly, levodopa is transported by amino acid transporters, rather than the P-glycoprotein, a transporter that can be inhibited by BAs [25]. Thirdly, the association between metabolomics and LR may be resulting from other metabolites. Integrating microbiome and untargeted metabolomics data may provide more meaningful insights. More precise macro-metabolomic approaches are needed to reveal the potential association between gut microbial interactions and the treatment effect of levodopa. Additionally, impaired bioavailability and clinical response to levodopa may be affected by the physiological properties of the gastrointestinal tract factors, including pH, buffer capacity, epithelial permeability, and gastrointestinal motility [26].

Our findings should be interpreted in the context of the following limitations. First, the sample size of our study is too small to provide more potential possibility of associations. A larger sample size and an independent validation cohort are proposed in the future, which could provide more insights. Second, the association between metabolomics and LR resulting from other metabolites was not detected. Integrating microbiome and metabolomic data may derive more meaningful insights.

## 5. Conclusions

In conclusion, from our results, we did not find significant associations between fecal BAs and LR, except for a weak correlation with CDCA-3-β-glucuronide, which demonstrated little relationship between fecal BAs and levodopa’s therapeutic effect. However, research in this field, even with negative results, is essential to unlock the full potential of pharmacomicrobiomics for predicting patient responses and future precise therapeutic strategies for PD patients.

## Figures and Tables

**Table 1:** The demographic and clinical characteristics of patients with PD.

The Demographic and Clinical Characteristics	Total n = 92	Bottom 30% LR n = 27	Top 30% LR n = 28	*p*-Value
Male (%)	41 (44.57)	17 (62.96)	7 (25.00)	0.005
Age (years)	67.77 ± 6.71	68.59 ± 6.59	66.11 ± 5.93	0.154
BMI (kg/m^2^)	23.67 ± 2.78	24.54 ± 2.66	22.65 ± 2.68	0.014
Age of onset (years)	61.24 ± 7.47	62.07 ± 8.26	58.82 ± 6.66	0.088
Disease duration (years)	6.53 ± 3.62	6.52 ± 3.64	7.29 ± 3.63	0.318
Hoehn and Yahr stage	2.33 ± 0.59	2.24 ± 0.53	2.29 ± 0.50	0.801
LEDD (mg)	538.91 ± 256.08	576.62 ± 258.86	526.94 ± 211.36	0.544
Clinical phenotype				
PIGD (n, %)	60 (65.22)	18 (66.67)	15 (53.57)	0.322
Challenge dose (mg)	263.01 ± 107.19	263.89 ± 108.44	265.42 ± 96.05	0.747
Off-state MDS-UPDRS part III	34.55 ± 11.67	35.89 ± 8.30	32.46 ± 11.10	0.053
Best on-state MDS-UPDRS part III	19.80 ± 7.89	24.33 ± 6.44	14.93 ± 5.62	<0.001
Change value of MDS-UPDRS part III	−14.75 ± 5.69	−11.56 ± 2.67	−17.54 ± 6.22	<0.001
LR (%)	43.27 ± 9.49	32.52 ± 4.27	54.08 ± 6.37	<0.001
Time to peak (min)	81.52 ± 23.67	76.67 ± 20.94	81.43 ± 17.99	0.186
Off-state plasma levodopa concentration (ng/mL)	16.69 ± 17.28	15.99 ± 22.48	14.43 ± 13.64	0.429
Best on-state plasma levodopa concentration (ng/mL)	174.05 ± 331.81	81.55 ± 71.04	240.22 ± 452.82	0.092
Change of plasma levodopa concentration (ng/mL)	157.36 ± 332.09	65.56 ± 68.48	225.79 ± 452.74	0.074
Change rate of plasma levodopa concentration (%)	1971.22 ± 3728.28	1191.75 ± 1470.73	3561.56 ± 5946.1	0.354

Data were shown as mean ± SD or frequency (percentage). Differences between groups were assessed using the chi-square test for categorical data and the Mann–Whitney U test for numerical data. Bottom 30% LR: the group of bottom 30% levodopa response; top 30% LR: the group of top 30% levodopa response.

## Data Availability

The clinical data that support the findings of this study are available from the corresponding author, upon reasonable request. Sequences generated and analyzed during this study are accessible from the National Center for Biotechnology Information (NCBI) under the accession code PRJNA1127293.

## Supplementary Materials

The following supporting information can be downloaded at: https://www.mdpi.com/article/10.3390/microorganisms12071432/s1, Figure S1: Alpha- and beta-diversity analyses; Table S1: The association between fecal BAs and levodopa response. Table S2: The abudance of bacterial species between the groups.

## Abbreviations

PD: Parkinson’s disease; LR, levodopa response; BMI, Body Mass Index; LEDD, levodopa equivalent daily dosage; PIGD, postural instability and gait difficulty; “off-state”, defined as the period when all anti-PD drugs were withdrawn for at least 12 h; MDS-UPDRS, Movement Disorder Society-Unified Parkinson’s Disease Rating Scale; best “on-state”, defined as the peak of levodopa benefit in the levodopa challenge test at hospital; “off-state” and best “on-state” MDS-UPDRS part III score were both evaluated in levodopa challenge test; time to peak, latency to reach the best “on-state” condition; CDCA-3-β-glucuronide, chenodeoxycholic acid-3-β-D-glucuronide; LCA 3-sulfate, lithocholic acid 3-sulfate; α-MCA, alpha-muricholic acid; β-MCA, beta-muricholic acid; DCA, deoxycholic acid; UCA, ursocholic acid; GHCA, glycohyocholic acid; 23-NDCA, 23-nordeoxycholic acid; TDCA, taurodeoxycholate acid; GCA, glycocholic acid; GUDCA, glycoursodeoxycholic acid; GCDCA, glycochenodeoxycholic acid; THDCA, taurohyodeoxycholic acid; TUDCA, tauroursodeoxycholic acid; TCA, taurocholic acid; TCDCA, taurochenodeoxycholic acid; iso-LCA, isolithocholic acid; LCA, lithocholic acid; 3-DH-CA, 3-dehydrocholic acid; CA, cholic acid; 12-KLCA, 12-ketolithocholic acid; ACA, apocholic acid; CDCA, chenodeoxycholic acid; 7-KDCA, 7-ketodeoxycholic acid; 7-KLCA, 7-ketolithocholic acid; GDCA, glycodeoxycholic acid; TLCA, taurolithocholic acid; allo-CA, allocholic acid; 3β-UDCA, 3β-ursodeoxycholic acid; DH-LCA, dehydrolithocholic acid; 3β-CA, 3β-cholic acid; HCA, hyocholic acid; MDCA, murideoxycholic acid; 12-KCDCA, 12-ketochenodeoxycholicacid; UDCA, ursodeoxycholic acid.

## References

[1] De Lau L.M., Breteler M.M. (2006). Epidemiology of Parkinson’s disease. Lancet Neurol..

[2] Braak H., Rüb U., Gai W.P., Del Tredici K. (2003). Idiopathic Parkinson’s disease: Possible routes by which vulnerable neuronal types may be subject to neuroinvasion by an unknown pathogen. J. Neural Transm..

[3] Lima I.S., Pêgo A.C., Martins A.C., Prada A.R., Barros J.T., Martins G., Gozzelino R. (2023). Gut Dysbiosis: A Target for Protective Interventions against Parkinson’s Disease. Microorganisms.

[4] Tan A.H., Lim S.Y., Lang A.E. (2022). The microbiome-gut-brain axis in Parkinson disease—From basic research to the clinic. Nat. Rev. Neurol..

[5] Sethi K. (2008). Levodopa unresponsive symptoms in Parkinson disease. Mov. Disord..

[6] Zhao Q., Chen Y., Huang W., Zhou H., Zhang W. (2023). Drug-microbiota interactions: An emerging priority for precision medicine. Signal Transduct. Target. Ther..

[7] Pierantozzi M., Pietroiusti A., Brusa L., Galati S., Stefani A., Lunardi G., Fedele E., Sancesario G., Bernardi G., Bergamaschi A. (2006). Helicobacter pylori eradication and l-dopa absorption in patients with PD and motor fluctuations. Neurology.

[8] Fasano A., Bove F., Gabrielli M., Petracca M., Zocco M.A., Ragazzoni E., Barbaro F., Piano C., Fortuna S., Tortora A. (2013). The role of small intestinal bacterial overgrowth in Parkinson’s disease. Mov. Disord..

[9] Zhang Y., He X., Mo C., Liu X., Li J., Yan Z., Qian Y., Lai Y., Xu S., Yang X. (2022). Association Between Microbial Tyrosine Decarboxylase Gene and Levodopa Responsiveness in Patients With Parkinson Disease. Neurology.

[10] Maini Rekdal V., Bess E.N., Bisanz J.E., Turnbaugh P.J., Balskus E.P. (2019). Discovery and inhibition of an interspecies gut bacterial pathway for Levodopa metabolism. Science.

[11] Zhang X., Han Y., Huang W., Jin M., Gao Z. (2021). The influence of the gut microbiota on the bioavailability of oral drugs. Acta Pharm. Sin. B.

[12] Kaddurah-Daouk R., Baillie R.A., Zhu H., Zeng Z.B., Wiest M.M., Nguyen U.T., Wojnoonski K., Watkins S.M., Trupp M., Krauss R.M. (2011). Enteric microbiome metabolites correlate with response to simvastatin treatment. PLoS ONE.

[13] Han B., Lv X., Liu G., Li S., Fan J., Chen L., Huang Z., Lin G., Xu X., Huang Z. (2023). Gut microbiota-related bile acid metabolism-FXR/TGR5 axis impacts the response to anti-α4β7-integrin therapy in humanized mice with colitis. Gut Microbes.

[14] Enright E.F., Griffin B.T., Gahan C., Joyce S.A. (2018). Microbiome-mediated bile acid modification: Role in intestinal drug absorption and metabolism. Pharmacol. Res..

[15] Wang S., Xu C., Liu H., Wei W., Zhou X., Qian H., Zhou L., Zhang H., Wu L., Zhu C. (2023). Connecting the Gut Microbiota and Neurodegenerative Diseases: The Role of Bile Acids. Mol. Neurobiol..

[16] Payne T., Appleby M., Buckley E., van Gelder L., Mullish B.H., Sassani M., Dunning M.J., Hernandez D., Scholz S.W., McNeill A. (2023). A Double-Blind, Randomized, Placebo-Controlled Trial of Ursodeoxycholic Acid (UDCA) in Parkinson’s Disease. Mov. Disord..

[17] Daniel S.E., Lees A.J. (1993). Parkinson’s Disease Society Brain Bank, London: Overview and research. J. Neural Transm. Suppl..

[18] Stebbins G.T., Goetz C.G., Burn D.J., Jankovic J., Khoo T.K., Tilley B.C. (2013). How to identify tremor dominant and postural instability/gait difficulty groups with the movement disorder society unified Parkinson’s disease rating scale: Comparison with the unified Parkinson’s disease rating scale. Mov. Disord..

[19] Tomlinson C.L., Stowe R., Patel S., Rick C., Gray R., Clarke C.E. (2010). Systematic review of levodopa dose equivalency reporting in Parkinson’s disease. Mov. Disord..

[20] Saranza G., Lang A.E. (2021). Levodopa challenge test: Indications, protocol, and guide. J. Neurol..

[21] Beckers M., Bloem B.R., Verbeek M.M. (2022). Mechanisms of peripheral levodopa resistance in Parkinson’s disease. NPJ Park. Dis..

[22] Bashyal S., Seo J.E., Choi Y.W., Lee S. (2021). Bile acid transporter-mediated oral absorption of insulin via hydrophobic ion-pairing approach. J. Control Release.

[23] Pavlović N., Goločorbin-Kon S., Ðanić M., Stanimirov B., Al-Salami H., Stankov K., Mikov M. (2018). Bile Acids and Their Derivatives as Potential Modifiers of Drug Release and Pharmacokinetic Profiles. Front. Pharmacol..

[24] Chen J., Farrell G.C. (1996). Bile acids produce a generalized reduction of the catalytic activity of cytochromes P450 and other hepatic microsomal enzymes in vitro: Relevance to drug metabolism in experimental cholestasis. J. Gastroenterol. Hepatol..

[25] Lopalco A., Cutrignelli A., Denora N., Lopedota A., Franco M., Laquintana V. (2018). Transferrin Functionalized Liposomes Loading Dopamine HCl: Development and Permeability Studies across an In Vitro Model of Human Blood-Brain Barrier. Nanomaterials.

[26] Vinarov Z., Abdallah M., Agundez J., Allegaert K., Basit A.W., Braeckmans M., Ceulemans J., Corsetti M., Griffin B.T., Grimm M. (2021). Impact of gastrointestinal tract variability on oral drug absorption and pharmacokinetics: An UNGAP review. Eur. J. Pharm. Sci..

