# Supplementary material for: Association between Fecal Bile Acids and Levodopa Response in Patients with Parkinson’s Disease
Source: Microorganisms. 2024 Jul 15;12(7):1432. doi: 10.3390/microorganisms12071432 (PMC11278915; doi:10.3390/microorganisms12071432)
Supplement: Supplementary file 1 [file microorganisms-12-01432-s001.zip › supplementary+material-revised.pdf]

Supplementary Material

Supplementary Information of Methods

Subjects

The other inclusion criteria were as follows: (1) voluntary agreement to participate in the study; (2) no use of antibiotics for 3 months; and (3) no use of immune-related drugs such as steroids for 3 months. The exclusion criteria were as follows: (1) secondary parkinsonism, atypical parkinsonism, Alzheimer's disease, or other central nervous system diseases; (2) celiac disease; (3) lactlose intolerance; (4) chronic pancreatitis; (5) other gastrointestinal tract absorption disorders; (6) history of gastrointestinal surgery (except appendectomy); (7) inflammatory bowel disease (Crohn's disease, ulcerative colitis); (8) history of surgical or medical treatment for cancer within the last 3 years; (9) prebiotics and probiotic dietary; (10) metabolic diseases; and (11) investigator judgement that the candidate was not suited to participate in the study.

Plasma Levodopa Concentrations Analysis

LC-MS/MS was conducted to measure plasma levodopa concentrations using the TSQ Vantage triple quadrupole mass spectrometers (Thermo Fisher Scientific, MA, USA). A volume of 200 μL plasma sample was analyzed with levodopa (L-DOPA, RING-D3, 98%; Cambridge Isotope Laboratories Inc., MA, USA) as an internal standard (30 μL, 10 μg/mL); 50 μL of 40% acetonitrile trifluoroacetic acid solution was added, and the mixture was blended in a vortex for 1 min, and subsequently centrifuged at 15,000 × g for 20 min. The supernatant was injected to Waters ACQUITY UPLC HSS T3 column (150 mm × 4.6 mm, 1.7 μm) at a flow rate of 0.2 mL/min at 4 °C, and the mobile phase consisted of phase A (0.5% formic acid in water) and phase B (methanol). Mass spectrometry analysis was carried out in the positive ionization mode using select

reaction monitoring mode. The optimized conditions used for analysis were spray voltage of ± 3.5 KV, sheath gas pressure of 50 psi, aux gas pressure of 25 psi, and capillary temperature of 350 °C. Data was recorded and analyzed using Xcalibur MS Software (version 4.0; Thermo Fisher Scientific, MA, USA).

Microbiome Analysis

Fecal metagenomics from the groups with the lowest and highest tertile LR of PD patients were measured by the whole-metagenome shotgun sequencing carried out on the Illumina HiSeq4000 platform (Illumina Inc., San Diego, CA, United States) at Majorbio Bio-Pharm Technology Co., Ltd. (Shanghai, China). Total genomic DNA was extracted from fecal samples and the concentration and purity of total genomic DNA were determined by TBS-380 and NanoDrop2000. A total of 2,963,712,608 reads were generated. The raw data were analyzed firstly on the free online platform of Majorbio Cloud Platform (www.majorbio.com). Briefly, the raw sequencing reads were trimmed of adapters, and low-quality reads (length<50 bp or with a quality value <20 or having N bases) were removed by fastp (https://github.com/OpenGene/fastp, version 0.20.0). Reads were aligned to the human genome by BWA (http://bio-bwa.sourceforge.net, version 0.7.17) and any hit associated with the reads and their mated reads were removed. A total of 2,172,127,294 quality-filtered reads were left for the next step assembly. The quality-filtered data were assembled using MEGAHIT (https://github.com/voutcn/megahit, version 1.1.2). Contigs with a length ⩾ 300 bp were selected as the final assembling result. A non-redundant gene catalog was

constructed using CD-HIT (http://weizhongli-lab.org/cd-hit/, version 4.7) with 90% sequence identity and 90% coverage. Gene abundance for a certain sample was eatimated by SOAPaligner (https://github.com/ShujiaHuang/SOAPaligner, version soap2.21release) with 95% identity. A total of 4,182,392 genes were included in the non-redundant gene catalogue. Representative sequences were aligned with the NCBI non-redundant database for taxonomic identification using BLASTP (version 2.2.28+) with an e-value cutoff of 1e-5. Gene function prediction (Kyoto Encyclopedia of Genes and Genomes level 3) were fil-tered considering only pathways present in at least 20% of the samples with an e-value cut off of 1e-5. Differential abundance levels of bacterial taxa at species level and KEGG pathways with a Benjamini-Hochberg adjusted p-value of less than 0.05 were considered significant. alpha- and beta-diversity plots were obtained using the MicrobiomeAnalyst tool.

Supplement figure:

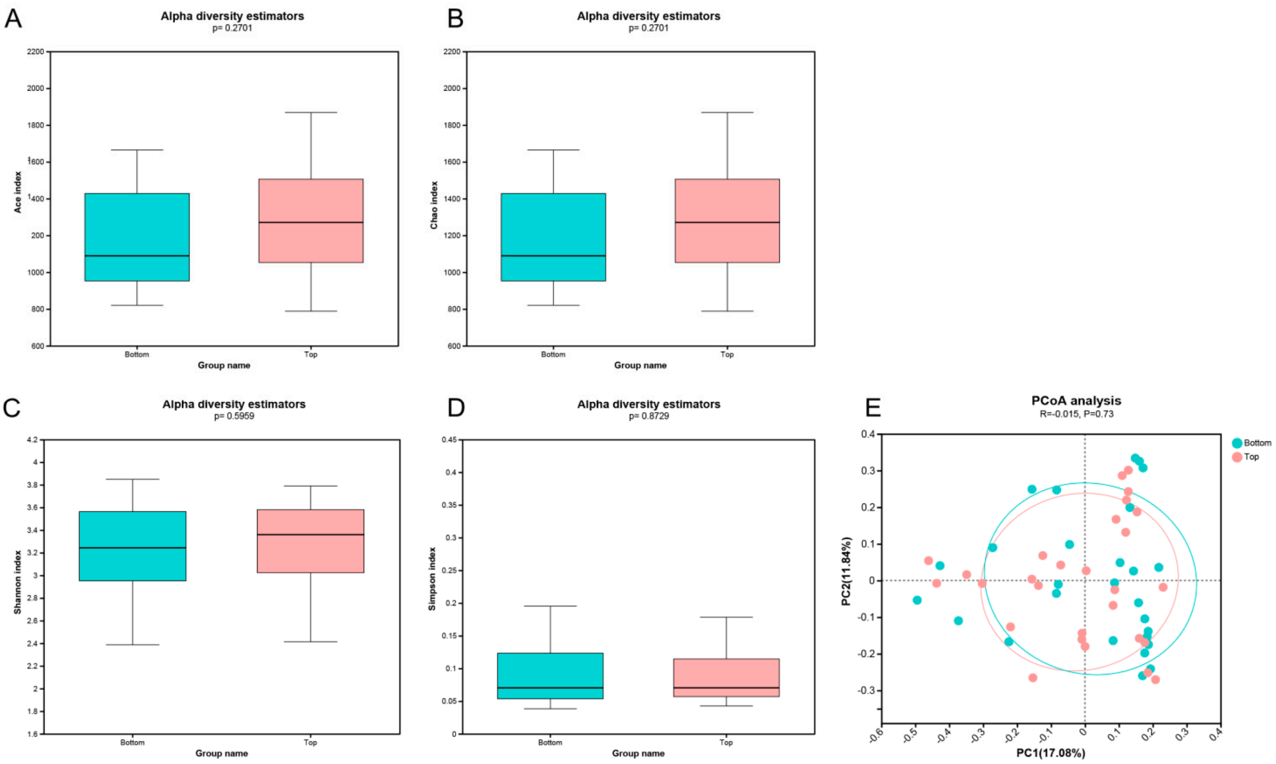

Figure S1: Alpha- and beta-diversity analyses at the species level between bottom and top 30% LR. (A-D) ACE, Chao, Shannon and Simpson indexes; (E) Principal coordinates analysis of Bray-Curtis distances.
